# Proteomics in Psoriasis

**DOI:** 10.3390/ijms20051141

**Published:** 2019-03-06

**Authors:** Leena Chularojanamontri, Norramon Charoenpipatsin, Narumol Silpa-Archa, Chanisada Wongpraparut, Visith Thongboonkerd

**Affiliations:** 1 Department of Dermatology, Faculty of Medicine Siriraj Hospital, Mahidol University, Bangkok 10700, Thailand; leenajim@gmail.com (L.C.); anonaemew@gmail.com (N.C.); doctornarumol@gmail.com (N.S.-A.); chanisada@hotmail.com (C.W.); 2 Medical Proteomics Unit, Office for Research and Development, Faculty of Medicine Siriraj Hospital, Mahidol University, Bangkok 10700, Thailand

**Keywords:** biomarker discovery, dermatology, diagnostics, prognostics, mass spectrometry, proteome, psoriatic skin

## Abstract

Psoriasis has been thought to be driven primarily by innate and adaptive immune systems that can be modified by genetic and environmental factors. Complex interplay between inflammatory cytokines and T-cells, especially Th1 and Th17 cells, leads to abnormal cell proliferation and psoriatic skin lesions. Nevertheless, such mechanisms do not entirely represent the pathogenesis of psoriasis. Moreover, earlier and better biomarkers in diagnostics, prognostics, and monitoring therapeutic outcomes of psoriasis are still needed. During the last two decades, proteomics (a systematic analysis of proteins for their identities, quantities, and functions) has been widely employed to psoriatic research. This review summarizes and discusses all of the previous studies that applied various modalities of proteomics technologies to psoriatic skin disease. The data obtained from such studies have led to (i) novel mechanisms and new hypotheses of the disease pathogenesis; (ii) biomarker discovery for diagnostics and prognostics; and (iii) proteome profiling for monitoring treatment efficacy and drug-induced toxicities.

## 1. Introduction

Psoriasis is a common, chronic, immune-mediated, inflammatory skin disease affecting humans worldwide with increasing prevalence and incidence, depending on geographical area [1]. The most common form is chronic plaque psoriasis, which is characterized by well-demarcated, erythematous plaques with silvery scales, accounting for approximately 85% of all psoriatic patients [2]. Although chronic plaque psoriasis can be found on all parts of the body, the most commonly affected areas include the elbows, knees, and scalp [3]. Psoriasis has been thought to be driven primarily by innate and adaptive immune systems that can be modified by genetic and environmental factors (e.g., alcohol, drugs, infections, skin trauma, smoking, and stress). The inflammatory cascade involves not only the skin but also other organs, leading to several comorbidities, e.g., psoriatic arthritis, metabolic syndrome (diabetes mellitus, hypertension, dyslipidemia, obesity, etc.), cardiovascular disease, non-alcoholic fatty liver disease, and kidney disorders [4]. Several lines of evidence have shown that T-cells, especially Th1 and Th17 cells, play crucial roles in such inflammatory responses [5]. However, antigen–antibody interactions also play important role, as demonstrated by the deposition of immunoglobulin G (IgG) and complement components in the upper epidermis of psoriatic skin [6]. Even with the aforementioned knowledge and advancements, the pathogenesis of psoriasis remains to be elucidated as there is a major part below the tip of the iceberg that is still covered, leading to unmet clinical needs [7]. In addition, current clinical practice in psoriasis for diagnostics, prognostics, and determination of the therapeutic outcome relies mainly on clinical findings and routine laboratory tests that frequently involve invasive procedures (i.e., skin and tissue biopsies) [7,8]. These limitations therefore warrant further investigations to better understand psoriatic pathogenesis and disease mechanisms, to define novel biomarkers for earlier and better diagnostics/prognostics, and to monitor treatment efficacy and drug-induced toxicities. 

During the last two decades, several advanced biotechnologies, e.g., omics-based technologies and molecular cell biology methods, have been widely applied to psoriasis to expand the knowledge of psoriatic pathogenesis and pathophysiology and to define novel biomarkers for diagnostics, prognostics, and monitoring treatment efficacy and drug-induced toxicities. Among these technologies, proteomics has been continuously developed and optimized for systematic analysis of proteins for their identities, quantities, and functions [9]. Based on its strength and capability, proteomics has been extensively employed for the investigations of psoriasis in several aspects. This review thoroughly summarizes all of the previous proteomics studies of psoriatic skin disease.

## 2. Brief Overview of Proteomics

Proteomics is a subdiscipline of protein science that relies mainly on the capabilities of protein separation techniques and protein identification (or profiling) by mass spectrometry [9,10]. In addition, genome information and bioinformatics are beneficial for further expanding the protein information to a broader extent and for guiding further functional investigations of the proteins of interest [11]. Commonly used methods of proteomics applied to biomedical research include two-dimensional gel electrophoresis (2-DE) followed by matrix-assisted laser desorption/ionization-time-of-flight mass spectrometry (MALDI-TOF MS), nanoflow liquid chromatography coupled with tandem mass spectrometry (nanoLC-MS/MS), surface-enhanced laser desorption/ionization time-of flight mass spectrometry (SELDI-TOF MS), capillary electrophoresis coupled with mass spectrometry (CE-MS), protein microarrays, and microfluidic technology on a chip [12,13]. Each technique has its own advantages and disadvantages and thus is complementary to the others. For example, 2-DE followed by MALDI-TOF MS and nanoLC-MS/MS can provide detailed protein information for readily functional investigations at subsequent stages but are generally time-consuming during analytical phase. In contrast, the latter approaches can be handled more easily and are more suitable for proteome profiling in clinical applications, i.e., for biomarker discovery to discriminate types of samples or to define biomarkers for diagnostics/prognostics. However, they are handicapped by the limited information of each protein in the whole proteome profile that needs more extensive investigations at later steps [9,10,12,13]. For all techniques, it should be noted that major abundant proteins in each biological sample (e.g., albumin in serum/plasma and keratin in skin samples) may be a major obstacle for analytical procedures (i.e., they can obscure identification and quantification of low abundant proteins). Therefore, elimination of these major abundant proteins may be required before proteomics analyses.

The data obtained from proteomics frequently lead to a better understanding of biology, physiology, and pathogenic mechanisms of diseases [14]. Additionally, proteomics also leads to biomarker discovery for disease diagnostics and prognostics as well as novel therapeutic targets [14,15,16]. With the same ultimate goals as for other diseases, all of the previous and recent proteomics studies of psoriasis are summarized in Table 1 and discussed as follows.

## 3. Proteomics Leads to Novel Mechanisms and New Hypotheses of the Psoriatic Pathogenesis

One of the strengths of proteomics is its ability to identify and characterize proteins that are involved in disease mechanisms in an unbiased manner without any need for prior information of such proteins in the disease model. Having done so, proteomics has led to several novel mechanisms and new hypotheses of the psoriatic pathogenesis. In 2005, Carlén et al. [17] employed 2-DE to differentiate proteome profiles of skins from patients with acute guttate psoriasis (associated with streptococcal throat infection), chronic plaque psoriasis, and nickel-induced contact eczema. The findings showed that the skin proteome profile of acute guttate psoriasis obviously differed from that of chronic plaque psoriasis, but was similar to that of contact eczema, indicating that the duration of the disease process may affect the skin proteome pattern [17]. Later, El-Rachkidy et al. [20] performed two-dimensional (2-D) immunoproteomics to examine components of the cellular proteome of *Streptococcus pyogenes* that were immunoreactive to blood circulating IgG from psoriatic patients. They have demonstrated that blood samples from patients with psoriasis contained significantly higher titers of IgG reactive to several elements of proteins from *S. pyogenes* than those from aged- and sex-matched healthy controls. These data indicate that *S. pyogenes* plays a more important role in the psoriatic pathogenesis/pathophysiology than we initially anticipated [20].

In a study by Plavina et al. [21], changes in levels of plasma glycoproteins and endogenous proteolytic activity in plasma samples collected from 20 psoriatic patients and 20 matched healthy controls were analyzed by glycoproteomics and peptidomics approaches using linear trap quadrupole (LTQ)-Fourier transform (FT)-nanoLC-MS/MS. The data showed that the proteins/peptides with the highest degree of increase in the psoriatic plasma were thymosin β4, followed by talin 1, actin γ, filamin, profilin, and calgranulins A and B. The increases in these cytoskeletal and actin-binding proteins/peptides as well as Ca^2+^-binding components have suggested disease-related cell leakage and altered protease activity in psoriasis [21]. Ryu et al. [23] further investigated proteins in lesional skin compared to non-lesional skin of 40 psoriatic patients and to the normal skin from five healthy individuals using 2-DE followed by nanoLC-MS/MS. The results demonstrated increased expression of several proteins, e.g., glutathione S transferase 1, peroxiredoxin 2, and SFN protein, in psoriatic lesional skin, indicating abnormalities in cell proliferation, the regulatory/balancing system, and the inflammatory response [23].

Using isobaric tags for relative and absolute quantification (iTRAQ) to quantitatively analyze proteins in epidermis, Schonthaler et al. [24] observed that S100A8, S100A9, and complement C3 were the three most up-regulated proteins in psoriatic lesional epidermis. Deletion of the gene encoding S100A9 could attenuate psoriasis-like skin disease and inflammation in a murine model [24]. Fattahi et al. [27] performed serum proteomics using 2-DE followed by MALDI-TOF/TOF MS/MS and identified abnormal expression of α-1-antitrypsin, keratin 10, and an unknown protein in the sera of patients with psoriasis that may lead to better understanding of the inflammatory process in psoriasis.

Lundberg et al. [28] used the KC-Tie2 murine model of psoriasis and screened for changes in the skin proteome by the label-free quantitative proteomics approach using LTQ-Orbitrap-nanoLC-MS/MS followed by validation in human samples. They highlighted the increases in kallikrein related peptidase 6, solute carrier family 25, cystatin A, and serpinB1 in psoriatic lesional skin. This study underscores the benefit of using an animal model in screening for changes in the skin proteome that finally led to identification of novel proteins involved in psoriasis [28]. Lysvand et al. [29] applied blue native gel electrophoresis and MALDI-TOF/TOF MS/MS to analyze protein complexes in the psoriatic scale and showed that post-translational modification (cleavage) of SerpinB3 (SCCA1) caused unique epitopes on the Pso p27 complex that may be responsible for the immunogenicity of such complex in psoriasis. Swindell et al. [30] utilized label-free, gel-enhanced LC-MS/MS (GeLC-MS/MS), LTQ-Orbitrap-nanoLC-MS/MS to compare protein expression in lesional vs. non-lesional skins from 14 psoriatic patients and found that 748 proteins had differential levels between the two groups, including those with concordant and discordant mRNA changes, most of which were targeted by interleukin-17A (IL-17A).

Recently, Bottoni et al. [31] utilized Fourier transform infrared (FT-IR) spectroscopy to analyze the saliva proteome and found that structural alterations of proteins in the saliva from patients with plaque psoriasis were similar to those of diabetic patients, both of which obviously differed from the normal saliva. However, the biological relevance to the disease mechanisms remained unknown and need further elucidations. Méhul et al. [37] applied labelled quantitative qTOF-MS/MS technology to compare the proteome of stratum corneum of lesional vs. non-lesional psoriatic skins. Quantitative analysis revealed differential levels of 140 proteins in these two areas, including those involved in the development of the epidermis, glycolysis, regulation of apoptosis, cytoskeletal organization, and peptide cross-linking, all of which may contribute to abnormal epidermal growth [37]. With antibodies-based techniques and protein array technology, the increased levels of chemoattractants of neutrophils, Th1 cells, monocytes, and dendritic cells were observed in psoriatic stratum corneum, whereas cytokines for Th2 cells that are known not to get involved in the disease pathophysiology were not found [37].

More recently, Gegotek et al. [40] applied GeLC-MS/MS based on LTQ-Orbitrap-nanoLC-MS/MS to analyze plasma samples from psoriatic patients compared to those obtained from sex- and age-matched healthy controls. Among several proteins identified, plasma from psoriatic patients had decreased levels of proteins involved in lipid metabolism and vitamin D regulation, whereas those involved in the immune response and signal transduction were increased [40]. While the involvement of proteins related to the immune response and signal transduction is not unexpected in the psoriatic pathophysiology, the decreased levels of proteins related to lipid metabolism and vitamin D regulation are quite interesting, although their precise roles in the disease mechanisms remain unclear.

Most recently, a study by Chularojanamontri et al. [39] applied a one-dimensional (1-D) immunoproteomics approach using the sera from 10 psoriatic patients compared to 10 normal sera to define autoantigens from the epidermis and dermis of normal skin, with the ultimate goal of a better understanding of the immune response at an early phase of the disease. The data obtained were quite interesting and showed that patterns of IgG- and IgM-immunoreactive autoantigens in both the epidermis and dermis as detected by psoriatic sera versus those detected by the normal sera were comparable without significant difference. These data indicate that the autoantigens derived from the epidermis and dermis of psoriatic skins and their humoral immune response are most likely the downstream event, whereas those associated with the upstream mechanism may be from cell-mediated immune mechanisms [39].

## 4. Proteomics Leads to Biomarker Discovery for Diagnostics and Prognostics in Psoriasis

Biomarker discovery is one of the major objectives for proteomics applied to psoriasis. Cowen et al. [19] used SELDI-TOF MS for proteome profiling of the sera from patients with psoriasis vulgaris and tumor-stage mycosis fungoides, as well as healthy controls. The data showed that serum proteome patterns of these three groups obviously differed and could differentiate the disease groups [19]. Williamson et al. [26] utilized a quantitative proteomics approach using stable isotope dimethyl labelling and LTQ-Orbitrap-nanoLC-MS/MS to screen for altered levels of proteins in psoriatic lesional skin as compared to the non-lesional area. Among the 50 differentially expressed proteins between groups, changes in levels of profilin 1, lysozyme C, and neutrophil gelatinase-associated lipocalin (NGAL) in the plasma were validated by selected reaction monitoring tandem mass spectrometry (SRM-MS/MS). However, only profilin 1 had a tendency to increase in the psoriatic plasma and thus may serve as a biomarker candidate for psoriasis [26].

Gschwandtner et al. [34] employed the label-free quantitative proteomics approach using GeLC-MS/MS to compare the mast cell proteome with that of the other skin cells (i.e., fibroblasts, keratinocytes, and melanocytes). The study revealed that two proteins, neural cell adhesion molecule L1 (L1CAM/CD171) and dipeptidyl peptidase 4 (DDP4/CD26), served as the novel biomarkers for human skin mast cells in normal, psoriatic, and mastocytosis skin [34]. Moreover, these two proteins may play crucial roles in skin homeostasis and their presence in mast cells may help elucidation of the unclear roles of mast cells in skin under normal and pathological states.

Matsuura et al. [36] applied MALDI-TOF MS and Triple-TOF MS/MS to profile serum proteomes and found that several peptides had differential levels in psoriatic sera versus controls. Among these, fibrinogen α chain-derived peptide and a filaggrin-derived peptide were consistently increased in patients with psoriasis vulgaris and psoriatic arthritis. Additionally, Wang et al. [38] employed aptamers-based protein microarray technology and found that kynureninase was distinctly increased in psoriatic sera and could differentiate psoriasis from atopic and contact dermatitis. Therefore, these serum peptides/proteins may serve as the novel biomarkers for psoriasis and/or new therapeutic targets [38].

To evaluate the disease severity, psoriasis and severity index (PASI) is the most commonly used score, which relies mainly on clinical parameters. However, the PASI score is somewhat problematic due to high degree of inter- and intra-individual variations of the examiners. There have been attempts to define more precise biomarkers that are correlated well with the disease severity. Reindl et al. [33] evaluated the plasma proteome profiles of psoriatic patients and healthy controls using label-free LTQ-Orbitrap-nanoLC-MS/MS method. Among 208 proteins whose levels were significantly altered in psoriatic plasma, changes in levels of desmoplakin, complement C3, polymeric immunoglobulin receptor, and cytokeratin 17 were correlated with the PASI score and thus may serve as the novel biomarkers for the disease severity [33].

## 5. Proteomics for Monitoring Treatment Efficacy in Psoriasis

Several targeted therapies and new modalities of treatment have been continuously developed according to the growing body of evidence for the pathogenesis of psoriasis. All of these new therapeutic strategies have the same ultimate goal to yield the highest efficacy with the least toxicity. Proteomics therefore can play role in monitoring treatment efficacy. Efalizumab, a previously available treatment for psoriasis, binds to CD11a and inhibits lymphocyte activation and cell migration [18]. Using multi-epitope ligand cartography (MELC) robot technology to analyze psoriatic skin after treatment, the data revealed a vast diversity of inflammatory epitope co-localization after efalizumab treatment of psoriasis [18]. However, it should be noted that efalizumab is no longer available on the market due to a risk of progressive multifocal leukoencephalopathy [41].

In addition, Kolbinger et al. [35] applied a targeted immunoproteomics approach to profile and quantify 170 proteins in skin and serum samples of eight psoriatic patients and eight healthy controls, before and after treatment with secukinumab (an anti-IL-17A monoclonal antibody). IL-17A was exclusively expressed in the lesional skin, not in the non-lesional area. Among several proteins examined, level of β-defensin 2 was correlated well with the IL-17A level and PASI score and was mostly increased in the lesional skins and sera of psoriatic patients [35]. After treatment with a single dose (300 mg) of secukinumab, levels of dysregulated antimicrobial peptides, proinflammatory cytokines, and neutrophil chemoattractants returned to normal. A significant reduction of β-defensin 2 was also observed [35]. Therefore, β-defensin 2 may serve as a biomarker for IL-17A-driven psoriatic pathology and for predicting response to the anti-IL-17A treatment.

Furthermore, Harvey et al. [32] used MALDI-MS imaging approach to analyze an engineered skin model generated by living skin equivalent (LSE) technology. Psoriasis was induced in this in vitro 3-D skin model by activation with the IL-22 inflammatory cytokine and was confirmed by histopathology and immunohistochemistry. MALDI-MS imaging was then employed to examine spatial distribution of acitretin (or psoriatane) in skin tissue after 48-h treatment. The data showed that acitretin was localized in the epidermis after 24-h treatment and then penetrated into the dermis after 48 h in both psoriasis and non-psoriasis models [32]. This may be considered as the first study in psoriatic research that demonstrates the feasibility of a proteomics approach to trace the skin tissue distribution of a therapeutic compound.

## 6. Proteomics for Monitoring Drug-Induced Toxicities in Psoriasis

Although novel targeted therapies for psoriasis can increase the therapeutic efficacy significantly, conventional treatments (e.g., using methotrexate, cyclosporine, and acitretin) are still widely used due to their cost-effectiveness. Nevertheless, long-term use of these conventional drugs, particularly methotrexate and cyclosporine, is associated with liver and renal toxicities, respectively. Tissue biopsy and histopathology are the gold standard methods to diagnose and monitor methotrexate-induced hepatotoxicity and cyclosporine-induced nephrotoxicity. However, tissue biopsy is an invasive procedure and may be associated with severe or fatal complications. Several alternative methods, including transient elastography (FibroScan), magnetic resonance elastography (MRE), and FibroTest, have thus been developed to monitor methotrexate-induced hepatotoxicity [42,43]. FibroScan is a vibrating device with an ultrasound probe that can create shear waves through the right lobe of the liver. The velocity of the shear wave is proportional to the degree of liver fibrosis [42]. It appears to have an ability to differentiate among normal liver, low-grade liver fibrosis, and high-grade liver fibrosis. Unfortunately, obesity (which is one of the most common comorbidities of psoriasis) is a major limitation for its use. Unlike FibroScan, MRE uses contrast magnetic resonance protocol to detect elasticity of the entire liver. Although MRE offers a high diagnostic accuracy, it is an expensive procedure and the data for its use to monitor methotrexate-induced liver fibrosis in psoriatic patients are quite limited. The principle for FibroTest is based on measuring serum levels of five known markers (γ-glutamyltranspeptidase, bilirubin, haptoglobin, apolipoprotein A-I, and α2-macroglobulin). Although it offers a sensitivity of 83% to screen for liver fibrosis, FibroTest is available only in some countries and there is a risk for false negatives in acute inflammation of the liver [42]. Therefore, searching for novel non-invasive biomarkers to detect and monitor drug-induced toxicities is required for the management of psoriasis.

Several attempts have been made to define such novel biomarkers using proteomics approaches. Van Swelm et al. [25] aimed to identify urinary biomarkers for methotrexate-induced hepatic fibrosis using MALDI-TOF MS and LTQ-nanoLC-MS/MS. Urine samples were collected from 60 psoriatic patients treated with low-cumulative dose (<1500 mg) or high-cumulative dose (>1500 mg) of methotrexate (note that liver fibrosis is more common in the latter group). Urinary proteome profiling showed that multiple proteins (i.e., N-cadherin, inter-α-trypsin inhibitor heavy chain H4, haptoglobin, and serotransferrin) may serve as the predictive urinary biomarkers for methotrexate-induced hepatic fibrosis [25]. Unfortunately, the reliability of these potential biomarkers was not validated with the gold standard (liver biopsy) or with other alternative methods such as FibroScan, MRE, and FibroTest.

Cyclosporine is a calcineurin inhibitor that is widely used for intermittent treatment of psoriasis to rapidly induce skin remission. Although not specific to psoriatic treatment, proteomics has been applied to search for potential biomarkers of cyclosporine-induced nephrotoxicity [22]. Lamoureux et al. [22] employed the stable isotope labeling with amino acids in cell culture (SILAC) approach followed by LC-MALDI-TOF/TOF MS/MS to investigate the effects of cyclosporine on the renal cellular proteome. As expected, levels of 69 proteins were significantly altered by cyclosporine, including those involved in the endoplasmic reticulum stress response, protein folding, apoptosis, metabolism, transport, cytoskeletal assembly, and nuclear/RNA regulation. Interestingly, administration of N-acetylcysteine, an antioxidant, could partially recover these proteome changes. Moreover, tacrolimus (another calcineurin inhibitor) caused changes in the renal cellular proteome that differed from those induced by cyclosporine [22]. Although not validated by in vivo study, these altered renal proteins may serve as potential biomarkers for cyclosporine-induced nephrotoxicity that should be further elucidated clinically and pathologically in psoriatic patients.

To our knowledge, there is no previous study that attempted to examine or monitor acitretin-induced toxicity by proteomics methodology. This may be due to its high level of safety, in which common adverse events affect only mucocutaneous tissue, whereas other side effects, i.e., hypertriglyceridemia, hypercholesterolemia, and transaminitis, can be monitored easily by blood chemistry.

## 7. Conclusions

During the past 13 years (since 2005), proteomics has been extensively applied to the investigations of psoriasis. With a wide range of proteomics methodologies, the data obtained from proteomics approaches have led to (i) novel mechanisms and new hypotheses of the disease pathogenesis; (ii) biomarker discovery for diagnostics and prognostics; and (iii) proteome profiling for monitoring treatment efficacy and drug-induced toxicities. Nevertheless, proteomics in psoriatic research is still in an early phase because there are still many gaps that need further investigations/elucidations. For example, validation of the candidate protein biomarkers in a large cohort of patients is warranted to precisely define diagnostic and prognostic biomarkers that can finally be used in clinical practice. Moreover, proteomics in combination with other emerging techniques will provide better understanding of the disease mechanisms and ultimately lead to the development of new therapeutic targets for the management of psoriasis in the future.

## Figures and Tables

**Table 1:** Summary of all proteomics studies in psoriasis.

Year	Authors/Reference	Proteomics Methodology	Sample(s)	Main Findings
2005	Carlén et al. [17]	2-DE, MALDI-TOF MS, Q-TOF-MS/MS	Skin	Proteome profile of chronic plaque psoriasis obviously differed from that of acute guttate psoriasis.
2007	Bonnekoh et al. [18]	Multi-epitope ligand cartography (MELC) robot technology	Skin	Topo-proteomic study using MELC robot technology showed a vast diversity of inflammatory epitope co-localization after efalizumab treatment of psoriasis.
2007	Cowen et al. [19]	SELDI-TOF MS	Serum	Serum proteomics could differentiate among subjects with neoplastic skin disease (mycosis fungoides), benign skin condition (psoriasis), and normal skin.
2007	El-Rachkidy et al. [20]	2-D immunoproteomics	Serum and plasma	Blood samples from patients with psoriasis contained significant higher titers of IgG reactive to the elements of proteins from *Streptococcus pyogenes* than those from aged- and sex-matched healthy controls.
2008	Plavina et al. [21]	Glycoproteomics, peptidomics, LTQ-FT-nanoLC-MS/MS	Plasma	Increased plasma levels of cytoskeletal and actin-binding proteins/peptides in psoriatic patients comparing to healthy controls.
2011	Lamoureux et al. [22]	SILAC, LC-MALDI-TOF/TOF MS/MS	HEK-293 renal cells	Levels of 69 proteins were significantly altered by cyclosporine and could be partially recovered by *N*-acetylcysteine, whereas the pattern of changes induced by tacrolimus obviously differed.
2011	Ryu et al. [23]	2-DE, nanoLC-MS/MS	Skin	Many proteins, e.g., glutathione S transferase 1, peroxiredoxin 2, and SFN protein, were increased in psoriatic lesional skin.
2013	Schonthaler et al. [24]	iTRAQ-2DLC-MS/MS	Epidermis	S100A8, S100A9, and complement C3 were the three most up-regulated proteins in psoriatic lesional epidermis. Deletion of the gene encoding S100A9 attenuated psoriasis-like skin disease and inflammation in a murine model.
2013	Van Swelm et al. [25]	MALDI-TOF MS, LTQ-nanoLC-MS/MS	Urine	Multiple proteins (i.e., N-cadherin, inter-α-trypsin inhibitor heavy chain H4, haptoglobin, and serotransferrin) may serve as the predictive urinary biomarkers for methotrexate-induced hepatic fibrosis.
2013	Williamson et al. [26]	Stable isotope dimethyl labelling, LTQ-Orbitrap-nanoLC-MS/MS, SRM-MS/MS	Skin and plasma	Over 50 proteins consistently differed in their expression levels in lesional vs. non-lesional psoriatic skins. Plasma profilin 1 may serve as a biomarker for psoriasis.
2014	Fattahi et al. [27]	2-DE, MALDI-TOF/TOF	Serum	Abnormal expression of α-1-antitrypsin, keratin 10 and an unknown protein in sera of patients with psoriasis.
2015	Lundberg et al. [28]	Label-free, LTQ-Orbitrap-nanoLC-MS/MS	Skin and primary keratinocytes	Kallikrein related peptidase 6, solute carrier family 25, cystatin A, and serpinB1 were increased in psoriatic lesional skin.
2015	Lysvand et al. [29]	Blue native gel electrophoresis, MALDI-TOF/TOF MS/MS	Stratum corneum	Post-translational modification (cleavage) of SerpinB3 (SCCA1) caused unique epitopes on the Pso p27 complex that may be responsible for the immunogenicity of such complex in psoriasis.
2015	Swindell et al. [30]	Label-free GeLC-MS/MS, LTQ-Orbitrap-nanoLC-MS/MS	Skin	748 proteins had differential levels between lesional and non-lesional biopsies, including those with concordant and discordant mRNA changes, most of which were targeted by IL-17A.
2016	Bottoni et al. [31]	FT-IR spectroscopy	Saliva	Structural alterations of proteins in saliva from patients with plaque psoriasis were similar to those of diabetic patients, both of which obviously differed from the normal saliva.
2016	Harvey et al. [32]	MALDI-MS imaging	Living skin equivalent (LSE)	Acitretin was localized in epidermis after 24-h treatment and then penetrated into dermis after 48-h in both psoriatic and nonpsoriatic models.
2016	Reindl et al. [33]	Label-free, LTQ-Orbitrap-nanoLC-MS/MS	Plasma	208 proteins had altered levels in psoriatic plasma. Among these, desmoplakin, complement C3, polymeric immunoglobulin receptor, and cytokeratin 17 might be used as biomarkers for disease severity.
2017	Gschwandtner et al. [34]	Label-free, GeLC-MS/MS	Skin mast cells, fibroblasts, keratinocytes, and melanocytes	L1CAM/CD171 and DPP4/CD26 serve as novel markers of human skin mast cells in normal, psoriasis, and mastocytosis skins and may be crucial for mast cell functions and skin homeostasis.
2017	Kolbinger et al. [35]	Targeted immunoproteomics	Serum and skin	B-defensin 2 was identified as a biomarker for IL-17A-driven psoriatic skin. Levels of dysregulated antimicrobial peptides, proinflammatory cytokines, and neutrophil chemoattractants returned to normal after secukinumab (anti-IL-17A) treatment.
2017	Matsuura et al. [36]	MALDI-TOF MS, TripleTOF-MS/MS	Serum	Several peptides had differential levels in psoriatic sera compared to controls. Among these, serum fibrinogen α chain-derived peptide and a filaggrin-derived peptide were consistently increased in patients with psoriasis vulgaris and psoriatic arthritis.
2017	Méhul et al. [37]	Protein array technology and qTOF-MS/MS	Stratum corneum and skin biopsies	140 proteins had altered levels in psoriatic stratum corneum, including those involved in the development of epidermis, glycolysis, regulation of apoptosis, cytoskeletal organization, and peptide cross-linking.
2017	Wang et al. [38]	Aptamers-based protein microarray technology	Serum	Kynureninase was distinctly increased in psoriatic sera and could differentiate psoriasis from atopic and contact dermatitis.
2018	Chularojanamontri et al. [39]	1-D immunoproteomics	Serum and skin	Humoral autoimmunity to epidermal and dermal autoantigens in psoriasis was most likely to be a downstream rather than an upstream effect.
2018	Gegotek et al. [40]	GeLC-MS/MS, LTQ Orbitrap-nanoLC-MS/MS	Plasma	Plasma from psoriatic patients had decreased levels of proteins involved in lipid metabolism and vitamin D regulation, whereas those involved in immune response and signal transduction were increased.

## Abbreviations

2-DE	two-dimensional gel electrophoresis
CE-MS	capillary electrophoresis coupled with mass spectrometry
FT	Fourier transform
FT-IR	Fourier transform infrared
GeLC-MS/MS	gel-enhanced liquid chromatography coupled with tandem mass spectrometry
IgG	immunoglobulin G
IL-17A	interleukin-17A
iTRAQ	isobaric tags for relative and absolute quantification
LTQ	linear trap quadrupole
LSE	living skin equivalent
MALDI-TOF MS	matrix-assisted laser desorption/ionization-time-of-flight mass spectrometry
MELC	multi-epitope ligand cartography
MRE	magnetic resonance elastography
nanoLC-MS/MS	nanoflow liquid chromatography coupled with tandem mass spectrometry
NGAL	neutrophil gelatinase-associated lipocalin
PASI	psoriasis and severity index
SELDI-TOF MS	surface-enhanced laser desorption/ionization time-of flight mass spectrometry
SILAC	stable isotope labeling with amino acids in cell culture
SRM-MS/MS	selected reaction monitoring tandem mass spectrometry
